# Assessing a set of optimal user interface parameters for intensity‐modulated proton therapy planning

**DOI:** 10.1120/jacmp.v11i4.3219

**Published:** 2010-08-20

**Authors:** Martin Hillbrand, Dietmar Georg

**Affiliations:** ^1^ Department for Radiotherapy, Division of Medical Radiation Physics Medical University of Vienna Vienna Austria

**Keywords:** proton beam planning, planning parameter, dose matrix resolution

## Abstract

The purpose was to identify an optimal set of treatment planning parameters and a minimal necessary dose matrix resolution for treatment planning with spot‐scanned protons. Treatment plans based on different combinations of planning parameters and dose grid resolutions (DG) were calculated in a homogeneous geometric phantom for three cubic targets of different size: 8, 64 and $244\;{\text{cm}}^{3}$. The proton dose was delivered by one single beam. Treatment plans were compared in terms of dose profiles parallel to and perpendicular to the central beam axis, as well as by dose homogeneity and conformity measures. Irrespective of target size, the dose homogeneity and conformity were comparable if the distance between spot layers was in the order of the width of a single Bragg peak, and the lateral distance between spots did not exceed two times the spot sigma. If the distance between spot layers was considerably larger than the width of the Bragg peak, the homogeneity index increased. For the small target, this index escalated from values around 5% to 12% in extreme, and to more than 20% for the two larger targets. Furthermore, the width of the 95% isodose increased. Similar results were found for the variation of the parameter determining the lateral spacing between proton dose spots. The average difference of dose profiles with respect to the profile for a DG of 1 mm was below 3% for all considered settings up to a DG of 6 mm. However, a DG of less than 2–3 mm is required to keep the maximum deviation below this limit. The tests performed in this study are necessary to prevent systematic errors from spot‐scanning proton therapy planning. A separation of dose spots in the dimensions of the Bragg peak in the longitudinal direction and no more than two times the spot sigma in the lateral direction were found to be adequate for IMPT treatment planning in a homogeneous phantom. A DG of 2–3 mm is necessary to accurately resolve the steep dose gradients of proton beams.

PACS number: 87.55.D‐; 7.55.de

## I. INTRODUCTION

The role of proton therapy in radiation oncology has increased during the last decade (i.e., its favorable depth dose distribution is explored in an increasing number of clinical and preclinical studies). In principle, two different modalities exist for proton beam delivery. Firstly, the narrow proton beam is spread to clinically relevant field sizes making use of (passive) scattering material. For the second group, a narrow pencil beam is actively scanned over the extent of the target volume. Most existing proton therapy centers are equipped with passively scattered proton beams. Spot‐scanned proton beams increase the potential to spare normal tissue located proximal to the target and enable the implementation of inversely planned intensity‐modulated proton therapy (IMPT) in a natural way. Therefore, most proton beam therapy facilities that were either recently built or are currently under construction are planned for active proton beam scanning technology.

Several dosimetric studies based on treatment planning comparisons were recently performed to benchmark proton therapy against advanced photon beam techniques.^(^ ^1^ ^–^ ^3^ ^)^ However, proton therapy planning is different compared to X‐ray–based therapy. Additional parameters, such as the proton range and uncertainties related thereto, have to be addressed. Previously published articles report on strategies how to account for such uncertainties.^(^ ^4^ ^–^ ^9^ ^)^ Particularly for spot‐scanning proton beams, the geometrical distribution of proton dose spots is a fundamental step in the treatment planning process. The total number of spots in the target is determined by the scanning volume parameters (i.e., geometrical parameters defining the lateral space between individual Bragg peaks, as well as the distance between spot layers).

The number of spots available for dose optimization reflects the number of degrees of freedom and, therefore, the ability to tailor the proton dose distribution to the individual needs of patient treatment. Due to practical reasons (e.g., the performance of the treatment planning hardware, the time necessary for proton dose calculation and the efficacy of treatment delivery), it is desirable to keep the total number of spots at a minimum level. Conversely, it is self‐evident that the distance between dose spots impacts on the characteristics of the target dose distribution (e.g., the dose homogeneity if the gap between individual spots increases excessively).

A similar tradeoff has to be found for the grid size of the dose calculation matrix. Due to steep dose gradients of particle beams – either laterally or distally to a single Bragg peak – an inhomogeneous proton dose distribution may fluctuate within only a few millimeters by several percentage points of the prescribed target dose. If the grid size of the dose calculation matrix is of the same order of magnitude or even less, these fluctuations might not appear in the final dose distribution due to interpolation artifacts.

The aim of this study was therefore to address these practical aspects of proton therapy planning, which are often overlooked and not scientifically evaluated. We claim that the tests described here are necessary prior to treatment planning in order to avoid systematic errors. In particular, the impact of the scanning volume parameters on the characteristics of the achievable dose distribution was determined, and an optimal setting of these parameters for IMPT treatment planning in a homogeneous geometrical phantom was identified. Beyond that, the minimal necessary resolution for the dose matrix reflecting an appropriate level of accuracy was intended to be determined.

## II. MATERIALS AND METHODS

### A. Treatment planning system and dose calculation

Treatment planning for spot‐scanning proton beams was performed on a 3D treatment planning system (XiO V4.34.0, Elekta CMS Software, St. Louis, MO). The treatment planning hardware was a Hewlett‐Packard XW 8000 workstation equipped with a Intel Xeon 3,0Ghz (dual core) CPU and 4GB of random access memory (RAM).

The pencil beam algorithm (and proton scatter calculation) for protons in the treatment planning system XiO was based on a publication by Soukup et al.^(^ ^10^ ^)^ In this work, the pencil beam model was tested against Monte Carlo simulations using Geant4 (Geant4 Collaboration, http://www.www.geant4.org). Furthermore, measured spot shapes were compared to calculated ones at several depths in a PMMA phantom during the dosimetric validation of the pencil beam algorithm. Elekta CMS Software reported an agreement of about 0.2 mm for all measurements (low and high energies), for all depths.^(^ ^11^ ^)^

At a given grid point of the dose calculation matrix, the contribution of all individual proton pencil beams, ${\text{PB}}_{i}$, within the range of three times the spot sigma are taken into account for dose calculation:

(1) $$D(x,y,z)=\sum_{PB_{i}\in [‐3\sigma ;+3\sigma ]}D_{CAX,i}(x_{0},y_{0},z_{0})\cdot F_{i}(x‐x_{0},y‐y_{0},z‐z_{0})$$

where $D_{\text{CAX},i}$ is the dose to be delivered by the i‐th pencil beam at its central axis (CAX) located at the coordinates $(x_{0}$,$y_{0}$,$z_{0})$. $F_{i}$ represents the relative fluence distribution of the considered pencil beam at the dose calculation point (x,y,z).

The initial shape of a proton dose spot was assumed to be a circular Gaussian,

(2) $$F(x,y,z)=\frac{1}{2\pi \sigma^{2}}\exp\left(‐\frac{{\left(x‐x_{0}\right)}^{2}}{2\sigma^{2}}‐\frac{{\left(y‐y_{0}\right)}^{2}}{2\sigma^{2}}\right)$$

where the z‐dependency of *F* arises from energy and depth dependent corrections for the spot sigma, $\underset{˙}{\sigma }=\sigma (z,\sigma_{0})$, due to multiple Coulomb scattering; $\sigma_{0}$ is the initial spot sigma in air. The Rossi approximation is used in XiO to derive scatter corrections to the spot sigma, which inherently limits the accuracy of dose calculations.^(^ ^12^ ^,^ ^13^ ^)^

The higher the expected grade of modulation in a beam, the higher are the accuracy requirements for dose calculation. The grade of modulation depends on the spot spacing and the prescription of the optimization problem. The fewer spots (degrees of freedom) available, the more modulation will be necessary to obtain satisfactory target dose conformity (or higher sparing of organs at risk). For a high grade of modulation, it is recommended to set the spots to a closer grid to improve dose calculation accuracy. The initial spot‐sigma, $\sigma_{0}$, was set at 3 mm in our particular beam model. Similarly, the width of the Bragg peak and therefore the thickness of the corresponding spot layer depend on the penetration depth due to scattering effects. For any specific particle energy, the peak width was derived from basic beam data measurements or by interpolation at depths where no measurements were available.

The distribution of proton spots over the target volume was arranged by an automatic function of the treatment planning system (TPS). Thereby, the spot layer with the maximal penetration depth coincides with the distal edge of the target volume. Based on this most distal spot layer, the remaining spots were uniformly distributed across the target volume according to constraints for the distance between spot layers and individual spots in the dimension perpendicular to the beam axis. These constraints are set by two user interface parameters – the spot spacing and the peak width multiplier (PWM). While the spot spacing is defined as an absolute distance given in millimeters, the longitudinal distance between individual spot layers is expressed in terms of multiples of the peak width for the respective particle energy. It therefore depends on the penetration depth. The peak width itself is defined as the width of the Bragg peak at 80% of the peak maximum.

### B. Geometrical phantom and treatment planning

**Figure 1:**
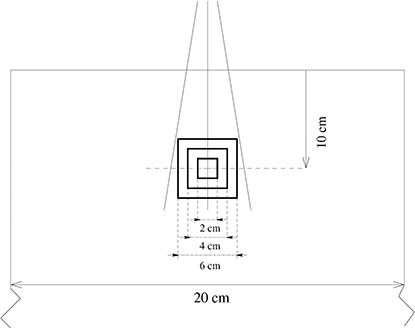
Schematic drawing of the homogeneous geometric phantom for treatment planning.

Treatment planning was performed on a cubic phantom (i.e., a non‐image based cubic structure). The dimensions of this structure were manually entered into the treatment planning system. A homogeneous relative electron density equal to water was assigned to the whole volume. The dimension of the outer contour was 20 cm, while individual slices were generated with 1 mm spacing in between. Three cubes of different sizes, namely 8, 64 and $216\;{\text{cm}}^{3}$, were created as virtual target structures. The target cubes were located concentrically at a depth of 10 cm and centrally with respect to the outer surface. A schematic drawing of the phantom is shown in Fig. 1.

For our purpose, a single constraint was set to achieve a homogeneous dose of 10 Gy to the target structure by a single intensity‐modulated proton beam (gantry angle at 0°). Spot weights were determined using a computerized optimization algorithm driven by physical dose‐volume constraints. Beam weight optimization was stopped if the convergence criterion of the gradient‐based optimization algorithm reached an accuracy of at least 0.0001 or after a maximum number of 100 iteration steps was reached. The “accuracy” during optimization in XiO is a relative number with respect to the cost function, where the initial dose distribution is represented by the value of 100. For a very few cases in our study, the optimization was stopped because the maximum number of 100 iterations was reached, but in any case the accuracy was then below 0.001. If necessary, the maximum number of iterations can be extended to 300 or a reoptimization (i.e., an optimization using a given fluence distribution as starting point) can be started.

The maximum available particle energy was 250 MeV corresponding to a maximum penetration depth in water of about 30 cm. Depth modulation was assumed to be achieved by energy degradation. The voxel size of the dose calculation grid was set to $1\times 1\times 1{\text{mm}}^{3}$, unless stated otherwise.

### C. Test conditions for scanning volume parameters and dose grid size

#### C.1 Peak width multiplier

Treatment plan optimization was run repeatedly varying the PWM from 0.4 to 2.0 in steps of 0.1. The spacing of the dose spots in lateral direction was set to 4 mm for this test.

To compare the different dose distributions, a depth dose profile was recorded along the central beam axis (CAX) and a homogeneity index (HI) (as defined in Eq. (3)) was evaluated within the extent of the target volume (i.e., between a depth of 9 to 11 cm, 8 to 12 cm and 7 to 13 cm for the small, medium and large target cube, respectively).

(3) $$HI(\%)=\frac{D_{\max}‐D_{\min}}{D_{\max}}\cdot 100$$

Furthermore, the width of the 95% isodose in the direction of the beam incidence was compared. This measure was considered as surrogate for the conformity index of the achieved dose distribution in the direction parallel to the beam incidence.

#### C.2 Lateral spacing of dose spots

An appropriate setting for the spot spacing was identified by a variation of the spotspacing parameter between 3 mm (congruent to one spot‐sigma) and 9 mm, in steps of 1 mm. Analogous to the PWM test, the HI index was evaluated within the extent of the target cube for a line profile through the center of the target and perpendicular to the central beam axis. Furthermore, the width of the 95% isodose was evaluated for these profiles.

#### C.3 Grid size of the dose calculation matrix

In order to investigate the impact of the dose matrix grid size (DG) on the output of the inverse planning and the characteristics of the achieved dose distribution, respectively, we have reoptimized the treatment plan for a fixed distribution of dose spots but changed the dose calculation grid size to between 1 to 6 mm, in steps of 1 mm. The PWM was 1.8 for the midsized target, and 2.0 for the two others as the fluctuations of the target dose were exceptionally pronounced for these two settings. Changes in the target dose homogeneity were recorded within the range of the target, but – in contrast to the variation of the PWM – reduced by 0.5 cm on both ends in order to exclude edge effects. Within the same depth range, the deviation of the profiles was calculated for all DG settings with respect to the profile corresponding to a DG of 1 mm (which we considered as the reference DG setting in our study).

## III. RESULTS

### A. Peak width multiplier

**Figure 2:**
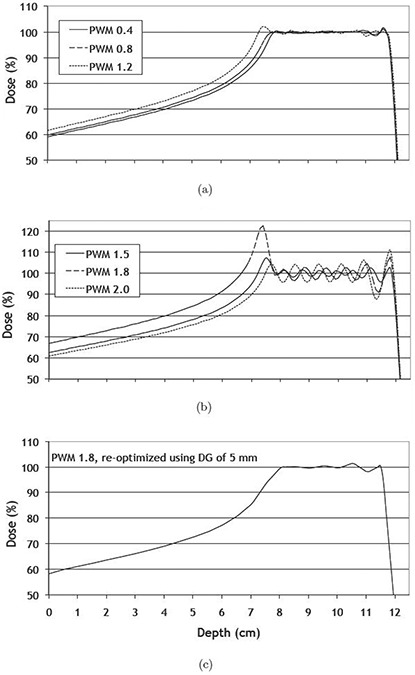
Dose profiles (a) along the central beam axis for the midsized target. The profiles for the small and the large target look similar and are therefore not shown. Panel (b) and (c) show both the dose profile for a PWM of 1.8. Note: in (c), the treatment plan was reoptimized using a dose grid resolution of 5 mm instead of 1 mm (as used in (b)).

The change of the target dose homogeneity as a function of increasing distance between spot layers is illustrated in Figs. 2(a) and (b), which show dose profiles for the midsized target. The plots for the small and the large targets look qualitatively the same and are therefore not presented. The size of the spot matrix was 17 by 17 for the large target, 13 by 13 for the midsized and 7 by 7 for the small target.

**Figure 3:**
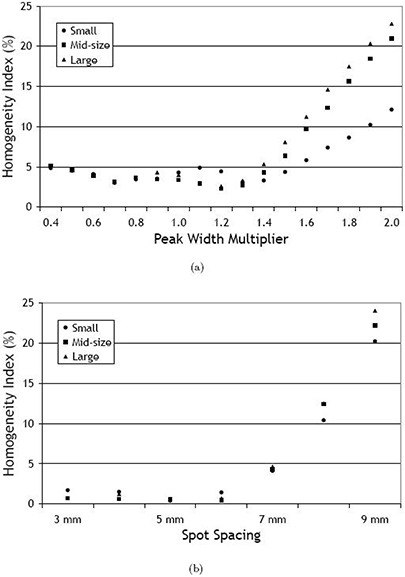
Target dose homogeneity for all three target sizes with respect to changes of the peak width multiplier (a) and to changes of the spot spacing (b).

Figure 3(a) shows the respective values of the HI. For the large target, no data was available for any PWM below 0.9, as for these settings the dose optimization failed by a shortage of random access memory. For this target, proton layers were located at depths between 13 and 7.06 cm, and the absolute separation of the spot layers ranged from 5.4 mm at the distal end to 3.7 mm at the proximal end of the target, for a PWM=1. For PWM values below or equal to 1.3, the HI varied between 2.5% and 5% for all three targets. If the distance between spot layers rises beyond 1.3, the HI increases. For example, for a PWM of 2.0, the dose distribution fluctuates by more than 20% with respect to the prescribed target dose.

The relative width of the 95% isodose with respect to the target size is listed in Table 1(a) for PMW values between 0.9 and 2.0. For any PWM below 1.1, the relative width was similar, around 1.05 to 1.15. Beyond that value, the dose conformity at the considered isodose level becomes worse and exceeds the width of the target by a maximum of 1.8 cm for a PWM of 2.0 (in the small target). Furthermore, the discrepancy between the width of the 95% isodose and the width of the target shows a periodic variation irrespective of target size. For every third to fourth setting of the PWM, the width of the 95% isodose achieves a local minimum. The magnitude of absolute deviations is comparable for all three target sizes. But, if evaluated relative to target size, these variations are most pronounced for the smallest target.

**Table 1:** Width of the 95% isodose relative to the width of the target.

a) Parallel to the central beam axis. Irrespective of target size, the relative size of the 95% isodose was nearly constant between 1.05 and 1.15 for all PWM below 1.0. These results are therefore not listed.											
*Peak Width Multiplier*											
	*1.0*	*1.1*	*1.2*	*1.3*	*1.4*	*1.5*	*1.6*	*1.7*	*1.8*	*1.9*	*2.0*
Small	1.05	1.15	1.30	1.10	1.20	1.40	1.50	1.15	1.25	1.35	1.80
Midsized	1.08	1.08	1.18	1.18	1.10	1.23	1.30	1.18	1.40	1.38	1.15
Large	1.08	1.03	1.05	1.07	1.17	1.13	1.07	1.20	1.08	1.07	0.95
b) Perpendicular to the central beam axis. In case of the large target and a spot spacing of 3 mm the dose optimization was not possible as a consequence of insufficient random access memory.											
	*3 mm*		*4 mm*		*5 mm*	*Spot Spacing 6 mm*		*7 mm*	*8 mm*		*9 mm*
Small	1.10		1.10		1.30	1.10		1.40	1.10		1.10
Midsized	1.04		1.10		1.15	1.13		1.20	1.35		1.49
Large	N/A		1.03		1.10	1.10		1.17	1.13		1.30

### B. Spot spacing

**Figure 4:**
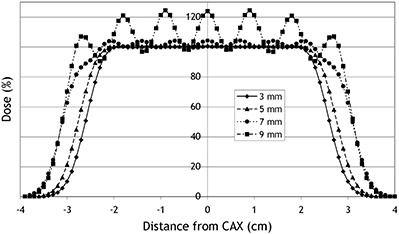
Dose profiles recorded perpendicular to the central axis for a spot spacing of 3, 5, 7 and 9 mm.

For all three target sizes, changes of the HI are shown in Fig. 3(b) with respect to variations of the spot spacing parameter. The relative width of the 95% isodose is summarized in Table 1(b). Some of the underlying dose profiles are shown in Fig. 4 – but only for the midsized target, as the profiles for the small and the large target were qualitatively similar. Furthermore, the data point for a spot spacing of 3 mm was not available in the case of the large target because the dose optimization for this parameter combination failed as a result of insufficient RAM.

Similarly to the variation of the PWM, a limit was found for the increment of the spot spacing parameter. For any spot spacing up to 6 mm (i.e., two times the initial spot sigma), the target dose homogeneity was around 0.5%–2%. Beyond this value, the HI increased and was 20%–25% for the largest spot spacing of 9 mm.

The width of the 95% isodose did not vary considerably for a spot spacing up to 6 mm in the case of the large and the midsized target, and for a spot spacing up to 4 mm for the small target. When the spot spacing exceeded these limits, the target dose conformity became worse. In extreme, the width of the 95% isodose exceeded the width of the target by almost 50% of the respective target size (e.g., for the midsized target and a spot spacing of 9 mm). With respect to the relative width of the 50% isodose (results not shown), similar results were also found for a spot spacing up to 6 mm. The width of the lateral penumbra – the distance between the 80% and 20% isodose level – was 5 mm irrespective of target size or the setting of the spot separation parameter.

### C. Grid size of the dose calculation matrix

For the reference dose matrix resolution of 1 mm, the HI index was 10.7%–13.1% in the central part of the target dose profile. For coarser DG resolutions, this index decreased almost linearly and was 0.1%–2.9% using a DG of 6 mm. The average and maximum deviations of the target dose are listed in Table 2 for all DG resolutions relative to the reference dose profile that were calculated with a DG of 1 mm. The average deviation was below 3% for all settings of the DG and for any target size. The maximum deviation was below 3% if the dose matrix resolution was less than 2–3 mm. However, in some cases the global dose maximum occurred outside of the target (e.g., for the midsized target and a PWM of 1.8 at a depth of 7.4 cm) where the peak dose was 122.5% using a DG of 1 mm (compare Fig. 2(b). When increasing the DG to 2 mm, the calculated peak dose decreased by 11% and, for any coarser dose matrix resolution, to values between 100%–110%. Using a DG of 5 mm, this peak disappeared in the dose profile, as shown in Fig. 2(c).

**Table 2:** Average and maximum deviation of the dose profiles relative to the profile for a dose grid of 1 mm.

	*Target Size*	*2 mm*	*3 mm*	*Dose Grid 4 mm*	*5 mm*	*6 mm*
Average (%)	Small	0.34	0.77	0.63	1.08	1.01
	Midsize	0.98	1.44	1.83	2.56	2.65
	Large	0.94	1.20	2.08	2.14	2.52
Maximum (%)	Small	1.38	3.06	1.72	4.39	3.55
	Midsize	2.60	5.59	3.78	7.88	9.08
	Large	2.49	3.05	4.92	3.97	6.25

Average and maximum deviation were recorded within the range of the target reduced by 0.5 cm on both ends in order to exclude edge effects.

### D. Time requirements for dose calculation and dose optimization

The time necessary for dose calculation and inverse dose optimization depends on the number of proton dose spots and the amount of calculation grid points, respectively.

It is important to note that the net time in minutes for dose calculations and optimization in an inverse treatment planning approach depends very much on the hardware available (i.e., the hardware is probably the largest factor of influence). During the end of this study, the hardware was upgraded from a dual‐core to a quad‐core HP workstation, which speeded all calculations by a factor of two. Therefore, time effects with respect to parameter settings are given in relative terms rather than absolute ones.

While the number of spots varies linearly with the parameters for the lateral spot spacing and the distance between spot layers, any change in the dose grid size results roughly in a cubic change of the time necessary for dose computation. For instance, the total time for dose calculation and dose optimization in the case of the large target volume was reduced by 55% (reduction from 40 min to less than 20 min) when the PWM was doubled from 1.0 to 2.0. The dose grid was set to 1 mm and the spot spacing to 4 mm for this run. For a fixed PWM of 1.0 and a spot spacing of 4 mm, this time was reduced to 13%, 4% and 1.8% of the original time for dose calculation when the DG was changed from 1 to 2, 3 and 4 mm, respectively.

## IV. DISCUSSION

Our results demonstrate that irrespective of target size, there are limits for the interspaces between proton dose spots in order to achieve clinically acceptable treatment plans. If these limits are exceeded, the weight of individual Bragg peaks has to be increased in excess to prevent cold spots within the target. Moreover, spots that were located outside of the target were assigned a dose equal or even higher than the target dose in order to obtain adequate dose coverage at the edge of the target. In case of high values of the PWM, a large dose weight was systematically assigned to such spots that were located proximally to the target. An example is shown in Fig. 2(b) for the midsized target, where the peak dose was 122.5% of the prescribed target dose for a spot located at 7.4 cm. If additionally the maximum dose in the unspecified tissue was constrained to less than the prescribed target dose, the peak dose decreased to 108% and the relative width of the 95% isodose from 1.40 to 1.28.

Furthermore, all these spots located proximally to the target caused periodic fluctuations of the target dose conformity. For all settings of the PWM larger than 1.1, the depth of these spots defined the proximal edge of the 95% isodose. At the distal end of the target, the position of the spots was per default fixed to the distal target edge. As a consequence, the distance between the shallowest spot layer and the proximal edge of the target became larger when the PWM increased and, furthermore, the dose conformity became worse. However, the treatment planning system restricts the location of the shallowest spot layer to an utmost extent of 1 cm proximally to the target. When this limit was exceeded, the whole layer was deleted. Then, the next layer that was closer to the edge of the target became the shallowest one and the dose conformity improved again.

All findings presented above were based on variations of the scanning volume parameters in a perfectly homogeneous cubic phantom. The limits for the spot spacing and the distance between spot layers can therefore not be easily transferred to all clinical situations. For the latter, a distortion of the spot matrix because of tissue inhomogeneities will prompt the demand for a higher number of spots to compensate for gaps. Such an increment of the total number of dose spots can be achieved by a reduction of the interspace between spots. On the other hand, clinically relevant treatment plans are usually based on more than a single beam, which also contributes additional spots for target dose optimization.

In our study, a fixed spot size of 3 mm was assumed. For high proton energies, spot sizes (Gaussian sigma) around 3 mm have been reported, those for lower energies are typically between 5 and 7 mm. With respect to the quality of a treatment plan, we would like to emphasize that besides the available spot sizes, the quality will depend very much on the optimization prescription, the number of beams used, the planning technique (single field uniform dose vs. full IMPT), and on practical issues that are associated with proton beam delivery.^(^ ^14^ ^,^ ^15^ ^)^ In a recent publication,^(^ ^15^ ^)^ it has been shown that constraints for the minimum deliverable monitor units and associated rounding errors could have a severe impact on the degradation of a spread out Bragg peak created by a scanning proton beam. As a consequence, a too small spot grid, where the number of spots bearing a very small spot weight is high, could be also disadvantageous in a realistic clinical situation.

The aim of the present study was to describe the dependency of the treatment plan quality on spot grid definition. The assumptions of a fixed spot size, a perfectly homogeneous geometric phantom and a single beam were made in order to avoid the influences from other factors on the characteristics of the dose distribution. Therefore we believe that the approach with a fixed initial spot sigma is reasonable for our theoretical study.

Kang et al.^(^ ^16^ ^)^ showed in their recently published study that the plan quality for a realistic clinical situation was almost identical for three different spot separation parameters (i.e., a uniform spot grid of 1 mm and 3 mm was compared to a nonuniform distance between spots of 0.5 times the Bragg Peak width). The authors stated that according to the Nyquist‐Shannon sampling theorem, a nonuniform spot separation in the dimensions of the Bragg peak width should have been adequate. Nevertheless, a separation of 0.5 times the Bragg peak width was chosen to compensate for deviations of the peak shape from an ideal Gaussian shape and to ensure adequate sampling. The method of using a nonuniform spot spacing corresponds exactly to the method of the Peak Width Multiplier, as used in the treatment planning system XiO. Thus, the findings by Kang et al. support our conclusion that a spot separation in the dimensions of the Bragg peak width is adequate in the absence of any distorting influence. In clinical situations, a finer spot grid is recommended to compensate for any distortion of the spot matrix.

The Nyquist‐Shannon sampling theorem was applied also by Li et al.^(^ ^17^ ^)^ to determine the minimum necessary dose matrix resolution. In this study, the theorem was applied to calculate the dose of a single Bragg peak within a 2% error limit at the Bragg peak point. As the width of the Bragg peak is broadened due to the depth dependent Coulomb scattering, the authors concluded that the maximum acceptable grid size enlarges with increasing depth. For penetration depths of 2 to 30 cm, they claimed a minimum necessary dose grid resolution of 0.4 to 6.8 mm for the demanded dose calculation accuracy of 2%. In particular, they found a maximum acceptable grid size of about 2 mm for a Bragg peak at a depth of approximately 7 cm. If these theoretical results are applied to our study, they particularly underline our finding that the dose in areas of steep dose gradients could only be accurately resolved if the DG was smaller than 2 mm (compare Fig. 2(c). In areas where the dose is homogeneous, we found that a DG of less than 4–5 mm is required in order to keep the average dose deviation with respect to the reference DG of 1 mm at about 2% or below.

Finally, it must be emphasized that fluctuations of the dose distribution by several percent of the prescribed target dose and within a few millimeters are a special concern of scanned particle beams, mainly as a consequence of their unique Bragg peak depth dose curve. These circumstances and our findings, therefore, stress the necessity for a small dose calculation grid in the case of IM proton beams.

## V. CONCLUSIONS

For any target size between 8 to $216\;{\text{cm}}^{3}$, a spot spacing of less then two times the spot sigma, $\sigma_{0}$, and a separation of the spot layers in the magnitude of the width of the Bragg peak for the considered depth, appear to be adequate for IMPT treatment planning in a homogeneous phantom. The average difference of dose profiles with respect to the profile for a DG of 1 mm was below 3% for all considered settings up to a DG of 6 mm. However, a DG of less than 2–3 mm is required to keep the maximum deviation below this limit.

## ACKNOWLEDGEMENTS

The authors want to acknowledge Dr. Gustav Meedt, CMS Elekta Softare, Freiburg, Germany, for his continuing support and valuable discussions.
